# A randomised cross over study to evaluate the performance of a novel ankle dorsiflexion measurement device for novice users

**DOI:** 10.1186/s13047-018-0286-x

**Published:** 2018-07-31

**Authors:** Peter R. Worsley, Caitlan Conington, Holly Stuart, Alice Patterson, Dan L. Bader

**Affiliations:** Clinical Academic Facility, Faculty of Health Sciences, University of Southampton, Southampton, SO17 1BJ UK

**Keywords:** Ankle dorsiflexion, Reliability, Goniometer, Inclinometer, Range of motion

## Abstract

**Background:**

The ankle joint is a common site of musculoskeletal pathology. Measurement of its functional range of motion is a primary indicator for rehabilitation outcomes in therapy settings. The present study was designed to assess reliability and validity of a new standardised method using a D-Flex device to assess ankle range of motion.

**Methods:**

A cohort of 20 healthy volunteers were recruited to measure the weight-bearing ankle range of motion using three assessment tools, namely, a goniometer, inclinometer and the D-Flex measurement devices. Repeated measures were performed both between and within observers for each device over a 48 h period. Performance evaluation of each device and their reliability was assessed using intra-class correlation coefficients and Bland and Altman plots.

**Results:**

Although significant correlations (*p* < 0.05) were observed between devices, there were large mean differences in ankle range of motion values ranging from 4.3°-15.7°. The D-flex produced the highest inter- and intra-rater reliability (ICCs 0.76–0.95), compared to values of 0.55–0.85 and 0.32–0.71 for the goniometer and inclinometer, respectively. The Bland and Altman plots revealed a low mean observer difference for the D-Flex (mean difference = 0.7°), with the vast majority of data coincident within the 95% confidence intervals. For both the goniometer and inclinometer mean differences were higher, with values of 3.1° and 5.7° respectively.

**Conclusion:**

The results of the present study provide evidence to support the use of the D-Flex system as a valid, portable, and easy to use alternative to the weight-bearing lunge test when assessing ankle dorsiflexion ROM in healthy participants.

## Background

The ankle is a common site for musculoskeletal injury for both the general public and specific sporting populations. Indeed, foot and ankle injuries are reported to represent over a quarter of musculoskeletal injuries in elite athletics [1]. Foot and ankle pathology is not isolated to young athletes, with a recent systematic review revealing pooled prevalence estimates for frequent foot pain of 24% and for frequent ankle pain of 15% [2]. Ankle sprains represent a common cause of ankle pain, accounting for between 3 and 5% of all Emergency Department visits in the UK, equating to approximately 5600 incidences per day [3]. Despite the high prevalence and potential severity of painful symptoms that follow the acute episode [4, 5], ankle sprains are commonly regarded as benign injuries that will resolve quickly with limited treatment [6, 7]. However, outcomes of conservative treatment range widely, with as many as 74% of patients with ankle sprains experiencing symptoms up to 4 years post injury [8]. Foot and ankle pathology typically results in pain, reduced range of motion and reduced quality of life [9, 10]. Restricted range of motion can affect mobility and participation in sports and social activity. Thus, improving range of motion in the joint is one of the primary goals of therapy.

Accurate measurement of joint range of motion (ROM) is necessary in both research and clinical settings [11]. Indeed, it is commonly used as an outcome measure to assess the efficacy of therapies [12]. The assessment of ROM during weight-bearing is considered to be most related to activities of daily living [13], replicating tasks such as stepping, walking and moving from sitting to standing. The evidence based standard for accurate measurement of ankle joint ROM involves radiographic measurement [14, 15]. However, this technique is not suitable for regular use in clinical practice for a number of reasons including exposure to radiation. This has resulted in the development of a number of non-invasive measurement approaches, although no one method can be regarded as the preferred methodology [16].

Goniometry is commonly used in the clinical setting to measure non-weight bearing ankle dorsiflexion ROM [16]. It involves the measurement of joint angles by an examiner, who places the arms of the device along the bones immediately proximal and distal to the joint, providing an estimated angle in degrees. However, evidence suggests that goniometry may not be a reliable method in either a research or clinical context [17, 18]. In addition, the technique requires a high degree of handling proficiency, with the starting position, centre of rotation and axis positions being prone to investigator error [19]. The inclinometers are also frequently used to assess weight-bearing ankle ROM, providing a digital display of inclination of the tibia bone relative to the ground [16]. Recently, inclinometer applications have been developed for a range of smartphones, which make them a popular choice to examiners. However, there is limited evidence regarding the validity and reliability of these devices [20]. Indeed, the Tiltmeter and iHandy represent the only validated smartphone applications to measure ankle dorsiflexion to date [11, 21].

There is a need to provide a standardised measurement technique for weight bearing ankle ROM measurement, with ease of use and portability are also critical factors for clinical translation. A new prototype device, known as the D-Flex, has been designed for application in any location, without the need for walls or flooring markings. The D-Flex provides a standardised method of ankle fixation with an automatic measurement system removing the potential for human error. However, there is no evidence regarding its performance when compared with goniometry or inclinometer devices. Accordingly, the study was designed to investigate the validity and reliability of D-Flex for the assessment of ankle ROM.

## Method

### Study design

This study used a randomised crossover design with repeated measures.

### Participants and setting

Participants were recruited from the local university population. Exclusion criteria included previous ankle pathology or surgery to the lower limb within the last 12 months, current illness or infection, neurological deficits affecting the lower limb and skin conditions or oedema affecting the lower limb. Full ethical approval was granted by the local institutional Ethics committee (ERGO-20696).

### Procedures

All testing was completed with the participants barefoot in a laboratory controlled at a temperature of 22 ± 2 °C. The end of range (EOR) dorsiflexion was measured using a weight-bearing lunge with first the knee flexed and then with the knee fully extended, which has been established as a method of assessment which is directly related to the functional activities performed in everyday life [19]. The participant initially stood with their feet parallel at hip width apart and subsequently performed a lunge using their self-selected dominant limb, such that their knee was located over their toes to attain end of range (EOR) dorsiflexion. The participant held this position for three seconds, whilst the measures were taken simultaneously with the maximal values recorded. The participant then returned their leg to a neutral position, before repeating this procedure two further times. The process was then repeated with the knee fully extended, where the participant lunged forward on their non-dominant leg, keeping their dominant knee straight, moving into EOR dorsiflexion. To assess inter-rater reliability, the researcher then left the room, and a separate investigator repeated the data collection process, blinded to the previous findings. The participant then returned to the laboratory 48 h later, and the whole process was repeated by the first researcher, for intra-rater reliability assessment. A random number generator was used to select leg position (knee flexed or extended) and the order in which devices were tested (D-Flex, goniometer or inclinometer).

### Materials

**Fig. 1:**
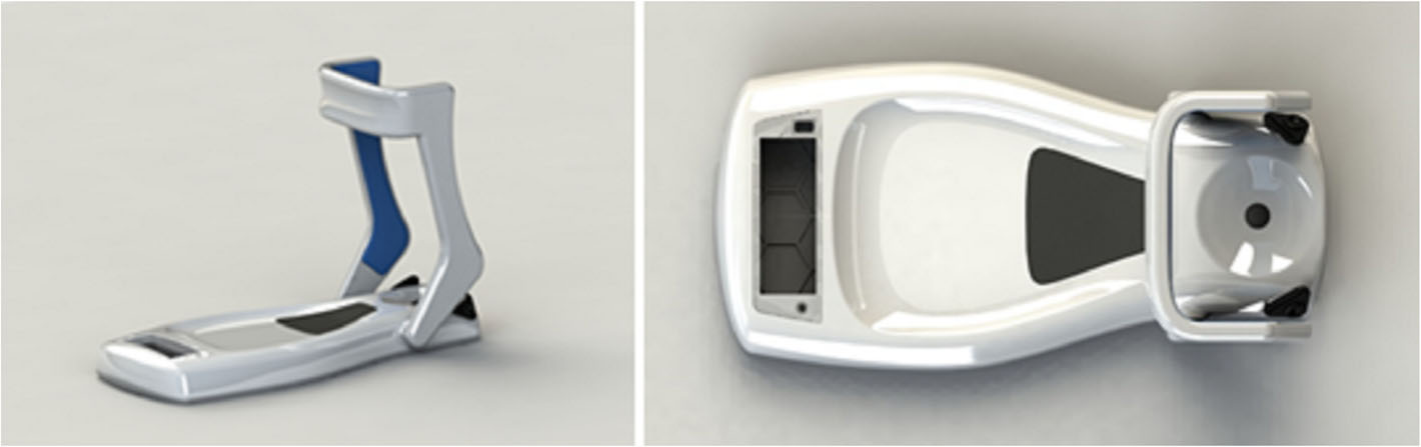
The D-Flex ankle dorsiflexion measurement device without talus strap

The D-Flex (Fig. 1) uses a standardised method of measurement to assess dorsiflexion ROM in the ankle. Velcro straps were applied inferior to the knee to fasten the lower limb into the device. This stabilised the foot in a precise position and isolated the ankle joint to prevent any unwanted movement. The D-Flex digitally calibrated when the foot was in plantar grade, and a green light indicated that the participant could move into end of range dorsiflexion. The participant was instructed to lean their knee over their toes to actively move into dorsiflexion, stopping when they reached the end of comfortable range. A force sensor under the heel detected when the foot was elevated off the measurement device as indicated by auditory feedback. The digital display was covered to blind the participants to minimise the risk of performance bias. Measurements were taken with and without a talus strap to assess for the effects of talocrural fixation.

**Fig. 2:**
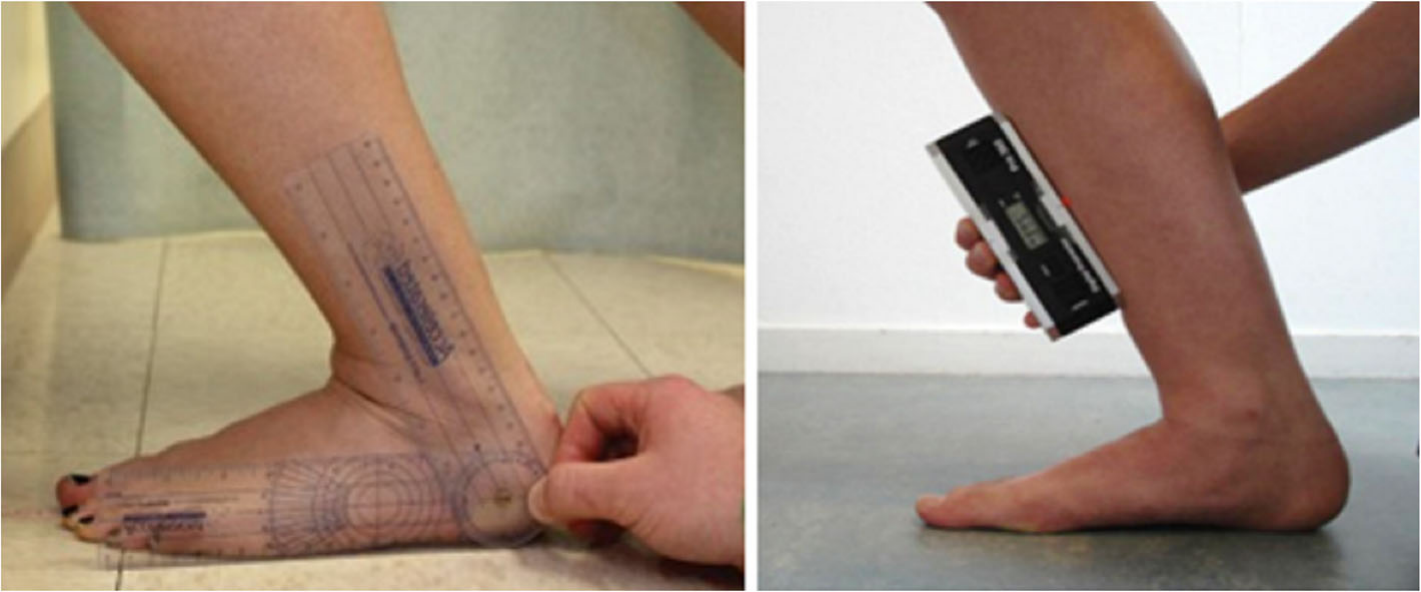
Goniometer (left) and Inclinometer (right) used in the present study

A universal goniometer and inclinometer were also used to measure dorsiflexion ROM (Fig. 2). Each were implemented with a standardised protocol [16]. The clear plastic goniometer (Protractor goniometer, Prestige, UK) was standard 7-in., flat, and measured in increments of 2°. The fulcrum was placed over the distal aspect of the lateral malleolus, and one arm was aligned with the head of the fibula. The moving arm was then aligned parallel with the fifth metatarsal of the foot, with this position representing zero degrees. A digital inclinometer measuring increments of 1° (Acumar Single Digital Inclinometer; Lafayette Instrument Company, Lafayette, IN) was placed on the tibial tuberosity and re-calibrated. A digital reading was then recorded from the device once the ankle had moved into EOR dorsiflexion.

### Statistical analysis:

Descriptive data involving means and standard deviation (SD) were calculated for each of the measurement techniques. The Intra-class Correlation Coefficient (ICC) and Bland and Altman tests were used to assess reliability. ICC models (2,1) and (1,1) were selected for inter- and intra-rater reliability, respectively, with associated 95% confidence intervals (CI) presented. Bland and Altman plots were created to visually represent bias and outliers, in conjunction with the degree of agreement between measures [22]. Reliability was defined as poor (ICC < 0.50), moderate (ICC 0.50 to 0.75), or good (ICC > 0.75) using previously established criteria [22]. Standard error of measurement (SEM) (SEM = SD √1-ICC) [23] and the minimal detectable change (MDC) (MDC=SEM * 1.96* √2) for each measurement technique were also calculated. Briefly, the SEM reflects absolute measurement error (response stability), and the MDC provides an objective threshold that can be used to determine whether values obtained are beyond measurement variability (i.e., smallest difference that can be accurately measured) [24]. The correlation between measurement devices was assessed using a Pearson’s correlation coefficient.

## Results

### Participants

A total of 20 participants were recruited from the local university population using poster advertisement. Participants included nine males and eleven females aged between 19 and 31 years old. Their mean (standard deviation) for height was 1.75 (0.1)m,weight was 69 (13.1)kg, with a corresponding mean BMI of 22.5 kg/m^2^.

Table 1 summarizes the measurements recorded by the three devices. There were clear differences between the measurements, with D-Flex recording the lowest ROM values and Inclinometer the highest (mean difference = 16.1°). The ROM in the ankle reduced with the knee fully extended, with values ranging between 10 and 35% depending on the measurement device.

**Table 1:** Mean and standard deviation (denoted in brackets) of ankle ROM values from three measurement devices

Leg position	D-Flex (no strap)	D-Flex (strap)	Goniometer	Inclinometer
Dorsiflexion with the knee flexed	18.2 (4.4)	17.5 (5.0)	22.5 (6.6)	33.9 (7.2)
Dorsiflexion with the knee straight	11.4 (3.0)	11.3 (3.5)	18.8 (5.6)	30.3 (7.7)

**Fig. 3:**
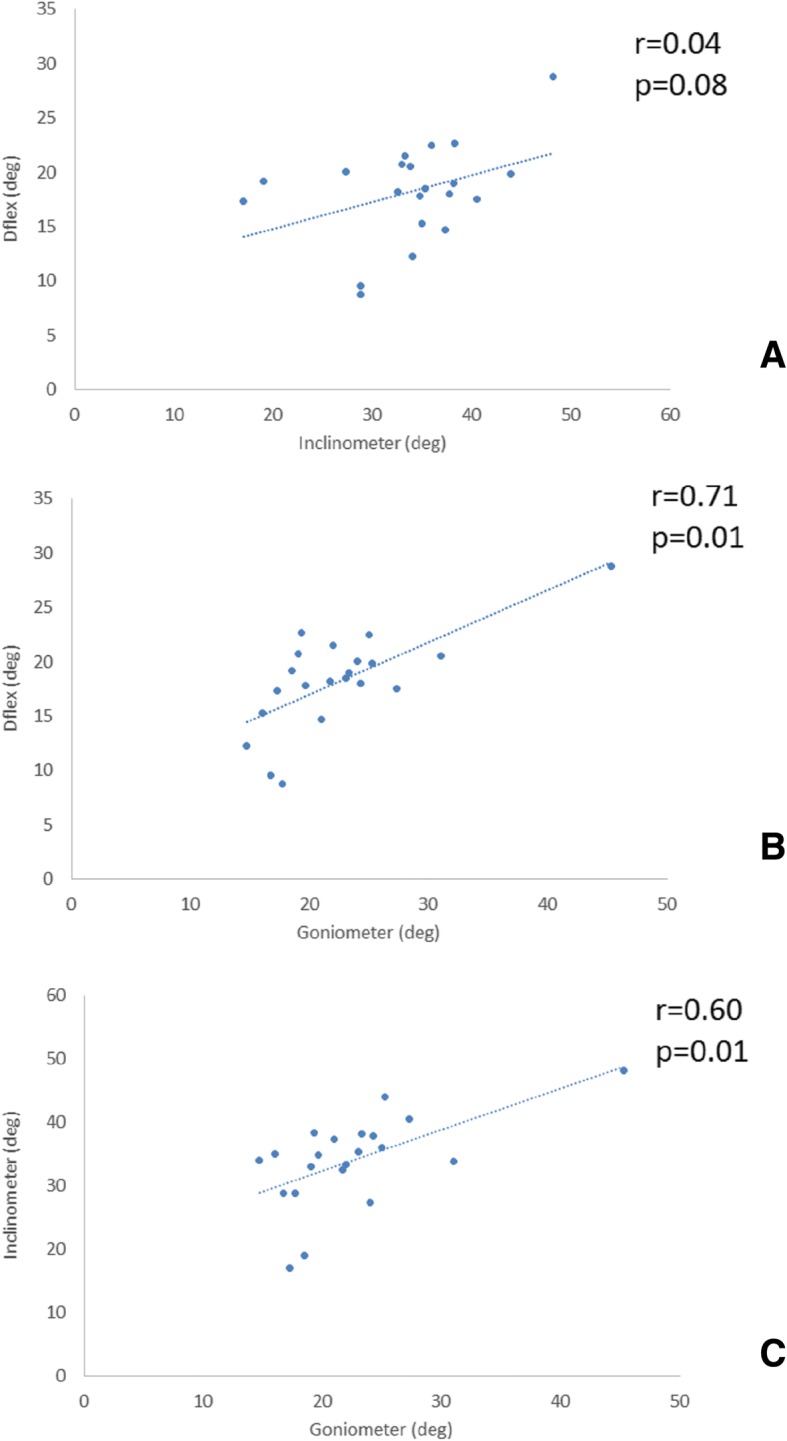
Correlations between the **a** goniometer and inclinometer, **b** goniometer and D-Flex and **c** inclinometer and D-Flex

The highest correlations between the measurement values was observed between the D-Flex and goniometer (*r* = 0.64–0.71, *p* < 0.001). The lowest values were between the inclinometer and the D-flex, with a corresponding r values =0.36–0.40, revealing a non-significant association (*p* > 0.05). Significant correlations (*p* < 0.01) between goniometer and inclinometer measures were observed with r values ranging from *r* = 0.56–0.6 (Fig. 3).

Inter-rater reliability of the D-flex was excellent under all test conditions, with all ICCs > 0.88. The goniometer also presented high levels of repeatability within observers with ICCs ranging between 0.75–0.85. The lowest level reliability was observed with the inclinometer, with the ICCs ranging between 0.41–0.71. (Tables 2 and 3).

**Table 2:** Inter-rater reliability results from the three measurement devices

		ICC	95% CI		*P* value	SEM	MDC
			Lower bound	Upper bound			
D-Flex	Knee flexion	0.95	0.87	0.98	< 0.01	0.88	2.44
	Knee extension	0.88	0.71	0.95	< 0.01	1.04	2.99
	Knee flexion (strap)	0.95	0.87	0.98	< 0.01	1.12	3.10
	Knee extension (strap)	0.92	0.81	0.97	< 0.01	0.99	2.74
Goniometer	Knee flexion	0.85	0.67	0.94	< 0.01	2.56	7.10
	Knee extension	0.75	0.47	0.89	< 0.01	2.8	7.76
Inclinometer	Knee flexion	0.41	0.03	0.71	0.32	5.53	15.3
	Knee extension	0.71	0.41	0.87	< 0.01	4.15	11.5

**Table 3:** Intra-rater reliability results from the three measurement devices

		ICC	95% Confidence intervals		*P* value
			Lower bound	Upper bound	
D-Flex	Knee flexion (no strap)	0.90	0.77	0.96	< 0.01
	Knee extension (no strap)	0.87	0.70	0.95	< 0.01
	Knee flexion (strap)	0.90	0.77	0.96	< 0.01
	Knee extension (strap)	0.76	0.49	0.90	< 0.01
Goniometer	Knee flexion	0.55	0.13	0.77	< 0.01
	Knee extension	0.61	0.26	0.89	< 0.01
Inclinometer	Knee flexion	0.32	0.11	0.60	0.44
	Knee extension	0.61	0.49	0.88	< 0.01

Results from the intra-rater assessment revealed lower reliability scores compared to the inter-rater. ICCs for the D-flex were again the highest values, ranging between 0.76–0.90. The goniometer and inclinometer had poor to moderate reliability with ICC scores of 0.55–0.61 and 0.32–0.61, respectively. The measurements of ankle ROM with the knee flexed using the inclinometer had the lowest reliability scores for both inter- and intra-rater assessments.

**Fig. 4:**
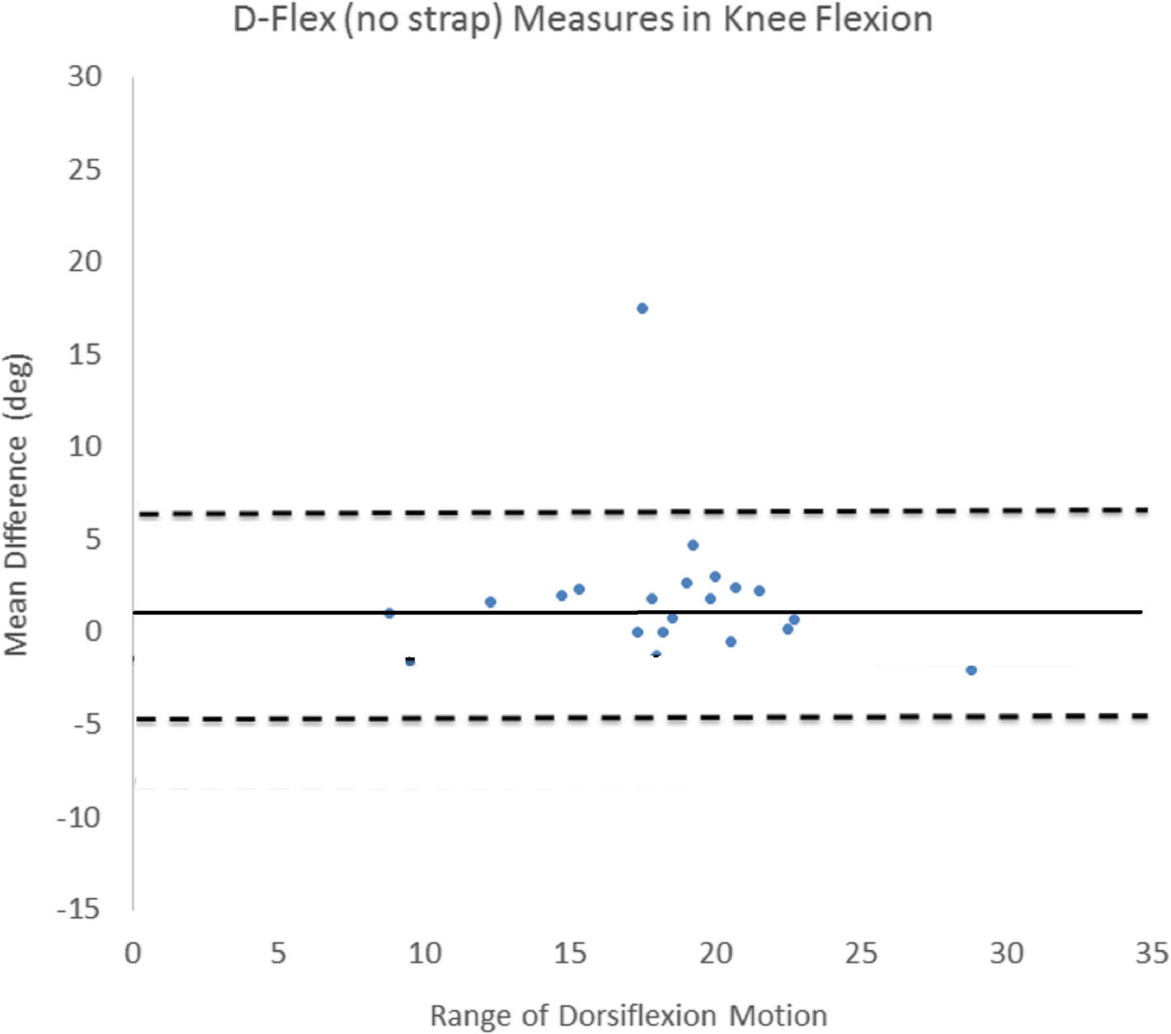
Bland and Altman plot for the inter-rater D-flex assessment with the talus strap and in knee fully extended

Figure 4 shows the Bland-Altman

plots for the inter-rater D-flex assessment with the talus strap and the knee fully extended. It indicates a low mean difference between raters (mean difference = 0.7°), with the vast majority of the data occurring within the 95% confidence intervals. There was no systematic bias between raters. For both the goniometer and inclinometer, mean differences were higher than the results achieved by the D-Flex. Inter-rater mean differences were 2.8–3.1 ^o^ and 4.4–5.7 ^o^ for goniometer and inclinometer, respectively. Furthermore, the confidence intervals for the goniometer and inclinometer were higher (> 15 degrees) than the corresponding values for the D-Flex (< 10 degrees).

## Discussion

The study aimed to assess the reliability and validity of a novel ankle dorsiflexion measurement device, the D-Flex, in comparison to conventional goniometer or inclinometer systems. Findings revealed that the D-Flex demonstratied both excellent inter and intra-rater reliability. However, the measures of ankle ROM from the D-Flex were much lower than those observed from the Goniometer and inclinometer. This could have been due to the design of the device, with a foot and ankle support isolating tibiotalar movements. Indeed, the order of magnitude in movement is similar to that observed in radiographic assessments of tibiotalar ROM [14] and other devices such as the Achillometer® device which also constrain the mid and forefoot during measurement [25].

The results of this study indicated that the D-Flex was a more reliable device to measure dorsiflexion ROM than the goniometer and inclinometer. This could have been a consequence of the relatively novice investigators using the devices. While it is difficult to compare between reliability coefficients from different studies, the weight-bearing tests provide the same or higher ICC values when they are compared with other tests using the inclinometer or the goniometer [16]. Goniometric intra-rater reliability is more widely accepted among clinicians, although results vary considerably between studies [18]. For example, Konor et al. (2012) reported very good intra-reliability (ICC = 0.85) correlating with the present results (ICC = 0.85, 0.75) [16], while by contrast, Popoff et al. (2012) reported poor to good intra-rater reliability (ICC = 0.50–0.75) [25]. This discrepancy may be explained by the fact that goniometry is dependent on the experience of the examiner due to the degree of technical proficiency involved [19]. The D-Flex revealed good to excellent intra-rater reliability (ICC = 0.76–0.90). Although this was the first study to examine the performance of the D-Flex, it is anticipated that future studies would maintain the high standard of reliability, due to the device’s standardised procedure and decreased risk of human error. The standard test method, ergonomic design and biofeedback (heel pressure) provide an ideal means for reliable testing across a range of experienced raters.

This standardisation of measurement protocol was a significant factor in the high inter-rater reliability of the D-Flex devices (ICC 0.88–0.95). These reliability values were higher than that of a digital inclinometer (ICC ranging from 0.77 to 0.88), when authors compared novice and experienced raters [26, 27]. The low measurement error for the D-Flex (SEM = 0.9–1.1°) is comparable to that of other newly designed dorsiflexion devices which have been examined [28, 29]. Although limited comparison can be made due to the varying metrics of measurement, these low measurement error values are in accordance with the values provided by Konor and colleagues (intra-rater SEM ranging from 40 to 60 mm) and Bennell and colleagues (intra-rater SEM ranging from 50 to 60 mm) for measurements taken using the tape measure.

The results of this study are limited to the healthy participants, so the results may not be extrapolated directly to injured populations. Indeed, further research using participants with ankle pathologies will establish whether the D-Flex could prove a beneficial tool for those patients with ROM deficits. The use of a rater with basic experience could be a limitation, particularly when evaluating reliability. However, the present data does infer highly reliable results that are similar to previous literature demonstrating that an individual with basic experience may perform the test with high reliability. It should be noted that the D-Flex relies on battery life. In addition, measures from the D-flex typically took three seconds, which could problematic for individuals with pain or pathology. The translation of the device will also be cost dependent, to date manufacture has limited to a few prototypes.

The differences observed between measurement devices were greater than values reported to be clinically relevant i.e. greater than minimal detectable change (MDC) values of ~ 5° [16, 30]. The low measures observed in the D-Flex will inevitably create a ceiling effect regarding the detection of clinically relevant changes in ankle dorsiflexion. Indeed, our estimated MDC values for D-Flex ranged between 2.4–3.1°. The corresponding MDC values for goniometry (ranging from 7.1–7.8°) are similar to those reported in the literature [16, 31]. However, the MDC values estimated for inclinometer assessment (11.5–15.3°) were far greater than values previously reported by Konner et al. (3.7–3.8°). This could be due to the difference in the experience of the raters and the technology used during the assessment.

## Conclusion

The present study has revealed that the D-Flex is a highly reliable method of measurement for ankle ROM, with very good intra-rater reliability, and excellent inter-rater reliability. In addition, it was identified as a more reliable device than both the universal goniometer and inclinometer, which are currently used in practice. It is anticipated that the D-Flex will prove a superior measurement tool for ankle dorsiflexion ROM, due to its ergonomic design and biofeedback mechanisms.

## Abbreviations

EOR: End of range
ICC: Intra-class Correlation Coefficient
MDC: Minimal detectable change
ROM: Range of Motion
SEM: Standard error of measurement
